# Clinical Significance and Therapeutic Potential of Long Non‐Coding RNA H19 in Soft Tissue Sarcoma

**DOI:** 10.1002/cam4.71305

**Published:** 2026-01-11

**Authors:** Stephan Jahn, Katarina Krajina, Maria Anna Smolle, Dimyana Neufeldt, Katharina Jonas, Beate Rinner, Kevin Mellert, Maxim Noeparast, Martin Trepel, Joanna Szkandera, Martin Pichler, Bernadette Liegl‐Azwanger

**Affiliations:** ^1^ Diagnostic and Research Institute of Pathology, Comprehensive Cancer Center, Subunit Sarcoma Medical University of Graz Graz Austria; ^2^ Translational Oncology, II. Med Clinics University of Augsburg Augsburg Germany; ^3^ InLEC – Interdisciplinary Laboratory for Experimental Cancer Research University of Augsburg Augsburg Germany; ^4^ Research Unit for Non‐Coding RNA and Genome Editing, Division of Oncology Medical University of Graz Graz Austria; ^5^ Department of Orthopaedics and Trauma Medical University of Graz Graz Austria; ^6^ Division of Biomedical Research Medical University of Graz Graz Austria; ^7^ Institute of Pathology University Hospital Ulm Ulm Germany; ^8^ Department of Hematology and Medical Oncology University Medical Center Augsburg Augsburg Germany; ^9^ Division of Oncology, Department of Internal Medicine Medical University of Graz Graz Austria; ^10^ Department of Oncology, Hematology and Palliative Care Clinics Oberwart Oberwart Austria

**Keywords:** H19, long non‐coding RNA, soft tissue sarcoma

## Abstract

**Background:**

Long non‐coding RNA (lncRNA) H19 plays a pivotal role in the pathogenesis of different human cancers, but its role in soft tissue sarcoma (STS) has not yet been defined.

**Methods:**

We analyzed H19 expression patterns in various cancer cell lines, focusing on sarcoma subtypes. RNA in situ hybridization was performed on a tissue microarray (*n* = 150) to assess H19 expression in human STS samples. Univariate and multivariate analyses were conducted to evaluate H19's prognostic value. In STS cell lines with high H19 expression, a Gapmer‐based knock‐down approach was used to study the functional impact of H19 expression.

**Results:**

Low H19 expression was associated with poor prognosis in univariate analysis (HR: 0.564; 95% CI: 0.324–0.985; *p* = 0.044). Multivariate analysis showed advanced patient age (*p* < 0.001) and large tumor size (*p* = 0.002) as independent predictors of worse overall survival, irrespective of H19 expression (HR: 0.655; 95% CI: 0.367–1.170; *p* = 0.153). H19 knockdown in STS cell lines reduced cellular growth and increased pro‐apoptotic activity.

**Discussion:**

Our findings suggest that H19 might play a role in STS pathogenesis. While its prognostic value requires further investigation, H19‐based targeting approaches may warrant evaluation for therapeutic potential in STS.

## 1 Introduction

Sarcoma is a rare form of malignancy originating from bone or soft tissue, and it represents a heterogeneous entity encompassing over a hundred histological subtypes, with soft tissue sarcoma (STS) being the most prevalent [1]. The diverse nature and rarity make it challenging to predict sarcomas' clinical consequences and develop novel treatment approaches [2]. Despite the low incidence, thousands of cases are diagnosed annually, often at advanced stages [3]. The 5‐year survival rate ranges strongly dependent on clinicopathological characteristics, declining sharply in advanced disease stages [4]. Further research is warranted to enhance our comprehension of the molecular pathogenesis of STS, identify reliable biomarkers and therapeutic targets, and translate these findings into effective treatment strategies [5].

Most of the human transcriptome encodes for non‐coding RNAs, and only a tiny fraction of approximately 2% encodes for proteins [6]. lncRNAs emerge as a distinct class of RNAs originating from non‐coding regions that are not translated into proteins but possess crucial regulatory functions with significant influence on carcinogenesis [7, 8, 9, 10]. Notably, lncRNAs are dysregulated in various types of cancer with the potential to serve as clinical biomarkers in multiple facets of cancer progression, either as oncogenic drivers or tumor suppressors [11, 12, 13].

H19, one of the first lncRNAs discovered, is located in the maternally imprinted region of chromosome 11 (11p15.5) and plays a crucial role in both human development and disease [14, 15]. Besides its role in genetic imprinting [16], H19 can act as a molecular decoy for microRNAs and as a molecular scaffold, affecting various growth and metabolism pathways [16].

The lncRNA H19 is frequently dysregulated in cancer and associated with tumor development and progression by influencing diverse molecular pathways encompassing cell proliferation, migration, invasion, apoptosis, and metastasis [17, 18, 19, 20]. Beyond its implication in tumorigenesis, H19 is involved in several biological processes, including epigenetic regulation and embryogenesis, as it is initially expressed in fetal tissues and decreases significantly after birth [21]. H19 is a so‐called oncofetal lncRNA with multifaceted functions [22]. Various factors tightly regulate and influence its expression, enabling it to possess oncogenic and tumor‐suppressive functions context‐dependently. Previous research has highlighted that H19 confers resistance to conventional therapies and promotes carcinogenesis through its oncogenic function in numerous cancers such as lung, bladder, breast, and gastrointestinal malignancies [18, 19, 23]. The molecular regulatory functions of H19 in soft tissue sarcoma are poorly understood. H19 and its imprinted neighbor IGF2 belong to the same cluster and are affected by alterations in DNA methylation within their differentially methylated regions [24]. The maternal copy of H19 and the paternal IGF2 gene can exhibit distinctive methylation states [25]. Rhabdomyosarcoma cells show a loss of imprinting due to aberrant methylation in this region, resulting in increased expression of IGF2 and downregulation of H19 and its growth‐suppressing miRNAs [26, 27, 28].

However, only a few studies have explored the clinical and biological role of H19 in soft tissue sarcoma pathogenesis, as almost all previous H19 studies focused on osteosarcoma [29, 30]. As the mechanisms of action of H19 in STS remain largely unknown, we can only speculate that mechanisms similar to those of other abovementioned sarcoma types might play a similar role in STS. Thus, our study aims to pioneer this area of research by comprehensively evaluating H19 in the development and progression of soft tissue sarcoma. To our knowledge, no study has been published focusing on the prognostic value combined with the biological relevance of H19 in soft tissue sarcoma.

## 2 Material and Methods

### 2.1 Overview

A comprehensive approach was utilized to investigate the expression and functional significance of H19 in sarcoma. Initially, an in silico analysis was performed using publicly available RNA‐seq datasets from CCLE, TCGA, and GTEx to compare H19 expression across multiple cancer types, including soft tissue sarcomas. These data provided a foundational reference for H19 expression patterns in various malignancies. To further validate and expand these findings, cell line‐based experiments were conducted. RNA was extracted from sarcoma cell lines, converted to cDNA, and analyzed via quantitative RT‐PCR to assess H19 expression levels relative to housekeeping genes.

Additionally, tissue microarrays (TMAs) were utilized to examine H19 expression at the tissue level through in situ hybridization, employing automated whole‐slide imaging and computational quantification to ensure objective signal detection. Computational tools were further used to quantify tumor cell nuclei and RNA point signals, providing a robust assessment of H19 transcript abundance per nucleus. To elucidate the functional role of H19, cell culture‐based transfection assays were performed using Gapmers to knockdown H19 expression in sarcoma cell lines. The effects of H19 silencing were analyzed through multiple assays, including cellular proliferation (WST‐1), clonogenic capacity (colony formation assay), apoptosis induction (Caspase 3/7 activity), and protein expression (Western blot analysis).

Lastly, statistical analyses were carried out to investigate the correlation between H19 expression levels and clinical outcomes. A combination of non‐parametric tests, regression models, and survival analyses (Kaplan–Meier and Cox regression) was employed to determine the prognostic significance of H19 expression in sarcoma.

### 2.2 In Silico Analysis

The online available RNA‐seq dataset provided by the Broad Institute Cancer Cell Line Encyclopedia (CCLE) was analyzed to compare H19 expression across multiple cancer cell lines (https://portals.broadinstitute.org/ccle). The visualization presents normalized expression data for H19 as box plots for cell lines from 38 different cancer types. The number of cell lines tested from each cancer type is given, and box plots are sorted according to the highest average expression within the cancer type, with the mean expression indicated as a dashed line. To compare H19 expression in tissue samples from different tumor types, data from The Cancer Genome Atlas Program (TCGA) and the Genotype‐Tissue Expression (GTEx) project were analyzed using the online tool GEPIA (http://gepia.cancer‐pku.cn/index.html). This data set comprised RNA‐seq expression data from 31 cancer types, including 262 soft tissue sarcoma tissue samples.

### 2.3 RNA Isolation and cDNA Synthesis From Cell Line Panels

Following the manufacturer's protocol, RNA was isolated from the sarcoma cell lines (at a confluency of approximately 75%–90%) in biological triplicates using TRIzol Reagent (Thermo Fisher Scientific, Waltham, MA, USA). From each sample, 1 μg of total RNA was reverse transcribed into cDNA after removal of genomic DNA by applying the QuantiTect Reverse Transcription Kit (Qiagen, Venlo, Netherlands) according to the manufacturer's protocol. We extended the incubation period of the reverse transcription at 42°C to 60 min.

### 2.4 Quantitative RT‐PCR for Cell Line Panels

Quantitative RT‐PCR was conducted with the synthesized cDNA to detect the expression levels of H19 in the sarcoma cell lines and was carried out in technical duplicates of the biological triplicates using the QuantiTect SYBR Green PCR Kit (Qiagen) according to the manufacturer's two‐step RT‐PCR protocol. Primers specific for H19 and the two housekeeper genes GAPDH and U6 were used (Eurofins Scientific, Vienna, Austria) at a final concentration of 0.4 μM. The primer sequences were: H19‐forward 5′‐TGC TGC ACT TTA CAA CCA CTG‐3′, H19‐reverse 5′‐ATG GTG TCT TTG ATG TTG GGC‐3′, GAPDH‐forward 5′‐AAG GTC GGA GTC AAC GGA TTT‐3′, GAPDH‐reverse 5′‐ACC AGA GTT AAA AGC AGC CCT G‐3′, U6‐forward 5′‐CTC GCT TCG GCA GCA CA‐3′, U6‐reverse 5′‐AAC GCT TCA CGA ATT TGC GT‐3′. Per reaction (volume of 10 μL), 10 ng of cDNA was used. The measurements were conducted in LightCycler 480 Multiwell Plates 384 on a LightCycler 480 Real‐Time PCR System (Roche, Basel, Switzerland). CT values were normalized by subtracting the according arithmetic mean of the two housekeeper genes. The received delta CT (dCT) values for each sarcoma cell line were plotted as 2^−dCT^ using GraphPad Prism Version 5.01 (GraphPad Software Inc., San Diego, CA, USA).

### 2.5 Tissue Microarrays (TMAs)

The tissue microarray was manufactured at the Institute of Pathology, Medical University of Graz, as previously described [31]. In total, 1846 cores from 334 patients were subjected to staining of H19 as described below. The study was approved by the local ethics commission of the Medical University of Graz (No. 29‐205 ex 16/17).

### 2.6 TMA Sectioning and H19 In Situ Hybridization

According to previous protocols [31], TMA FFPE Blocks were cut at 4 μm and mounted on positively charged slides (Superfrost Plus, ThermoFisher Scientific). RNA in situ hybridization staining reagents were purchased from Advanced Cell Diagnostics (ACD), Hayward, CA, USA RNAscope 2.5 HD Reagent Kit‐BROWN (cat.#: 32300) for FFPE was used as a detection system. Staining was performed according to the manufacturer's instructions. Slides were boiled in target retrieval solution for 15 min, and protease plus digestion was performed for 30 min at 40°C. Briefly, we used a horseradish peroxidase‐based signal amplification system for hybridization to the target probes, followed by color development with 3,3′‐diaminobenzidine. Finally, the slides were counterstained with hematoxylin for 10 s at room temperature. Brown punctate dots in the nucleus or cytoplasm determined positive staining. All RNAscope target probes were purchased from Advanced Cell Diagnostics, USA: Positive control probe Peptidylpropyl isomerase B (RNAScope Positive Control Probe Hs‐PPIB, cat.# 313901), negative control probe DapB (RNAScope Negative Control Probe DapB, cat.# 310043), H19 lncRNA target probe (RNAScope Probe Hs‐H19 cat.# 400771).

### 2.7 Acquisition of Whole Slide Images and Exclusion of Tissue Artifacts

Whole slide images (WSI) were acquired on a 3DHISTECH P1000 Scanner (3DHISTECH Ltd., Budapest, Hungary). We used extended focus mode and an 80‐fold magnification objective for image acquisition. WSI images were further processed with the TMA module of the 3DHISTECH‐Case‐Center suite. Single cores were automatically detected and manually vernier adjusted. Cores were inspected using the CaseViewer software (3DHISTECH). Automatically assigned regions of interest (ROI) designated for analysis were manually readjusted for exact location and shape, where necessary, to cover the relevant core and exclude artifact regions such as tissue folds and out‐of‐focus areas due to semi‐detached tissue fragments. Each core on the TMAs was assigned a unique tissue core ID (i.e., a pseudocode) mapped to the patient's tumor and linked to the clinical data.

### 2.8 Automated Quantification of Tumor Cell Nuclei and Stained lncRNA Point‐Signals

Cell boundaries are generally poorly demarcated in sarcomas. Accordingly, we used tumor cell nuclei counts as a surrogate for cell counting (one tumor nucleus = one cell). The 3DHistech Quant Center (module: Cell and Nucleus Detection Algorithm) was employed for counting tumor cell nuclei. The default settings were adjusted and calibrated by visual inspection of highlighted tumor cell nuclei called by the software. We noted that in high target lncRNA expressing tumors, tumor cell nuclei were obscured by superimposed, sometimes confluent, brown point signals, which precluded accurate calling of nuclei by the algorithm. Accordingly, tumor nuclei counts were evaluated on the corresponding core of the negative RNA control. The number of nuclei per ROI of the negative control was divided by the ROI area, resulting in a “nuclei per mm^2^” value. This value was then multiplied by the tumor area (ROI) of the respective lncRNA stain (H19, positive and negative control probe) to calculate the total amount of tumor cell nuclei in the ROI of the core.

The RNA‐Scope technique visualizes each RNA transcript with a single point signal, providing quantitative and linear RNA transcript evaluation. To count these point signals on the WSIs, we first employed the 3DHISTECH Quant Center software module dedicated to point‐signal detection. Results were satisfactory for well‐separated point signals but failed to adequately call areas of confluent signals seen in tumors with high transcript expression. Therefore, we used a pixel classifier for counting stained lncRNA transcripts: The number of brown pixels corresponding to all the signals from stained RNA transcripts in the ROI was divided by the average pixel size of a single transcript (set to 5 pixels/transcript) to calculate the total number of transcripts in the ROI per core.

The average number of target transcripts per cell was derived from the total number of transcripts called by the algorithm divided by the total number of tumor cell nuclei counted for each core. Thus, we obtained the average number of target transcripts per nucleus (ANTT) for H19 as well as for the positive and negative control probes in the ROI of the respective core. These values were subsequently used for statistical evaluation. After training the quantification models, the automated calling performance on 20 randomly chosen cores was compared to manual (visual) evaluation. TMA cores were visually evaluated for the ANTT from WSIs displayed on the 3DHISTECH case viewer software. The allocation to ANTT quartiles demonstrated a good correlation between manual and automated evaluation by the 3DHISTECH software.

TMA cores were excluded from further statistical evaluation due to insufficient RNA quality if ANTT was < 1 signal in the positive control probe PPIB and the target probe H19. TMA cores were also excluded in cases with ANTT > 1 in the negative control probe DapB.

### 2.9 Cell Culture for Transfection Approach

The sarcoma cell lines SW872 (Liposarcoma) and SW982 (Synovial Sarcoma) were purchased from the American Type Culture Collection (ATCC; Manassas, CA, USA). Both cell lines were maintained in DMEM: F12 containing 10% fetal bovine serum (FBS) (Serana, Pessin, Germany), 2 mM L‐Glutamine (Gibco, Thermo Fisher Scientific, Waltham, MA, USA), and 1% penicillin/streptomycin (Sigma‐Aldrich, St. Louis, MO, USA). All cell lines were cultivated at 37°C and under a 5% CO_2_ atmosphere and tested regularly for mycoplasma infection.

### 2.10 RNA Isolation and cDNA Synthesis Upon Gapmer Transfection

Total RNA was isolated from cultured cells (at a confluency of approximately 75%–90%) in biological triplicates using Qiazolzol Reagent (Thermo Fisher Scientific, Waltham, MA, USA) following the manufacturer's protocol. For each sample, 1 μg of total RNA was reverse transcribed into cDNA using the Biozym cDNA Synthesis Kit (Biozym Scientific GmbH, Germany) according to the manufacturer's protocol.

### 2.11 Quantitative RT‐PCR Upon Gapmer‐Transfection

To quantify expression levels of H19 in sarcoma cell lines, quantitative RT‐PCR was carried out in biological triplicates using PowerUp SYBR Green Master Mix (Applied Biosystems, ThermoFisher Scientific, Waltham, USA) according to the manufacturer's protocol (20 ng cDNA/10 μL reaction volume). Measurements were done on a QuantStudio 6/7 Pro real‐time PCR system (Applied Biosystems, ThermoFisher Scientific, Waltham, USA). Expression levels were normalized to the housekeeping genes TATA‐binding protein (TBP) and glyceraldehyde‐3‐phosphate dehydrogenase (GAPDH) and analyzed using the ΔΔCT method. Primers specific for H19 and for the housekeeping genes GAPDH and TBP were designed with the NIH Primer Blast Tool and purchased from Eurofins Scientific. Primer sequences are the following:

**Table:** 

Target gene	Forward	Reverse
H19	TGCTGCACTTTACAACCACTG	ATGGTGTCTTTGATGTTGGGC
TBP	TGCACAGGAGCCAAGAGTGAA	CACATCACAGCTCCCCACCA
GAPDH	AAGGTCGGAGTCAACGGATTT	ACCAGAGTTAAAAGCAGCCCTG

### 2.12 Gapmer‐Mediated KnockDown of H19

Gapmer‐mediated, transient knockdown of H19 was performed according to the fast‐forward protocol (Qiagen) using three different Gapmers with a final concentration of 20 nM (Gapmer #1, Gapmer #2, Gapmer #8; Qiagen, Hilden Germany). The Allstars Negative Control Gapmer A (Qiagen, Hilden, Germany) served as control. Experiments were performed 48 h after transfection.

### 2.13 WST‐1 Cellular Growth Assay

Cellular growth was assessed using the WST‐1 proliferation assay (Roche, Vienna, Austria). Cells were seeded in 96‐well plates at a density of 4 × 10^3^ cells and transfected with Gapmers against H19 using HiPerfect Transfection Reagent (Qiagen) according to the reverse transfection protocol (Qiagen). Cells were cultivated for 24–96 h, and the assay was conducted every 24 h. Therefore, 10 μL of the WST‐1 proliferation reagent was added per well and subsequently incubated for 1–2 h at 37°C. The absorbance was measured at 630 and 450 nm at each time point using a BioTek Synergy HTX Multimode Reader (Agilent, Santa Clara, CA, USA).

### 2.14 Colony Formation Assay

SW982 cells were seeded in 12‐well plates at a density of 1 × 10^5^ cells/well and transfected with Gapmers against H19 using the HiPerfect Transfection Reagent (Qiagen). 24 h after transfection, cells were trypsinized, counted, and seeded into 6‐well plates in triplicates at a density of 300 cells/well and incubated under standard conditions. Cells were fixed with 100% methanol and stained with 0.04% crystal violet solution (Sigma‐Aldrich) after 13 days. The number of colonies was counted manually using ImageJ software.

### 2.15 Caspase 3/7 Activity Assay

SW872 and SW982 cells were seeded in 96‐well plates at a density of 4 × 10^3^ cells/well and transfected with Gapmers for transient knockdown of H19 using HiPerfect Transfection Reagent (Qiagen) according to the reverse transfection protocol (Qiagen). The luminogenic substrate was added 48 h after transfection following the manufacturer's instructions and measured using a BioTek Synergy HTX Multimode Reader (Agilent, Santa Clara, CA, USA).

### 2.16 Protein Extraction and Western Blot

Cells were seeded into 6‐well plates with a density of 1.6 × 10^5^ cells/well and transfected with Gapmers for transient knockdown of H19 using the HiPerfect Transfection Reagent (Qiagen). 48 h after transfection, total protein was extracted using the radio‐immunoprecipitation assay (RIPA) buffer (Sigma‐Aldrich) supplemented with a 1:50 protease inhibitor cocktail (Sigma‐Aldrich). The protein concentration was quantified using the Pierce BCA Protein Assay Kit (#23225, Pierce, ThermoFisher Scientific, Washington, USA). 20 μg of total protein per sample was resuspended in Laemmli buffer (BioRad, Hercules, CA, USA) containing 10% β‐mercaptoethanol (Sigma‐Aldrich) and was heated at 95°C for 8 min. Proteins were separated by SDS‐PAGE utilizing 4%–15% Mini‐PROTEAN TGX Precast Gels (BioRad) and were subsequently transferred to nitrocellulose membranes (BioRad). The membranes were blocked in 5% non‐fat milk in 1× Tris‐buffered saline (TBS, BioRad)/0.1% Tween‐20 (Sigma‐Aldrich) for 1 h and then incubated with the primary antibodies diluted in 5% non‐fat milk at 4°C overnight. The following antibodies were used: apoptosis marker PARP (#9542, Cell Signaling Technology, Danvers, MA, USA, diluted in 1:1000) and β‐actin (#A5441, Sigma‐Aldrich, St. Louis, USA, diluted in 1:5000). After primary antibody incubation, the membranes were washed once with TBS‐Tween and were further incubated with the secondary HRP‐conjugated anti‐rabbit (#7074S, Cell Signaling Technology, Danvers, MA, USA) or anti‐mouse (#7076S, Cell Signaling Technology, Danvers, MA, USA) antibodies, respectively (both diluted 1:2000 in 5% milk). The signals were detected using an enhanced chemiluminescence detection system (SuperSignal Chemiluminescent Substrate, Thermo Fisher Scientific) and visualized on a BioRad ChemiDoc Touch device.

### 2.17 Statistical Analysis

All statistical analyses were carried out with Stata Version 16.1 (StataCorp, College Station, Texas, USA) and GraphPad Prism version 10.0.3 (GraphPad Software Inc., San Diego, CA, USA). Means and medians were provided with corresponding standard deviations and interquartile ranges, respectively. Mann–Whitney *U* and Kruskal–Wallis tests were used to analyze associations between H19 expression and binary or categorical variables. Logistic regression analysis was applied to assess associations between H19 expression and continuous variables. Univariate and multivariate Cox regression analyses for overall survival (OS) with death as an endpoint were performed to investigate potential prognostic factors. Kaplan–Meier curves were plotted for prognostic parameters to allow graphical visualization. Variables with significant associations in the univariate analysis were entered into the multivariate model. Values of bar graphs are expressed as the mean ± SD. To assess statistically significant differences between the two experimental conditions, an unpaired *t*‐test or one‐way ANOVA was applied where appropriate to calculate statistical differences between the control group and various treatment groups. *P* value below 0.05 was considered statistically significant (*p < 0.05, **p < 0.01, ***p < 0.001, ****p < 0.0001).

## 3 Results

### 3.1 H19 Is Expressed in Soft Tissue Sarcoma

**FIGURE 1:**
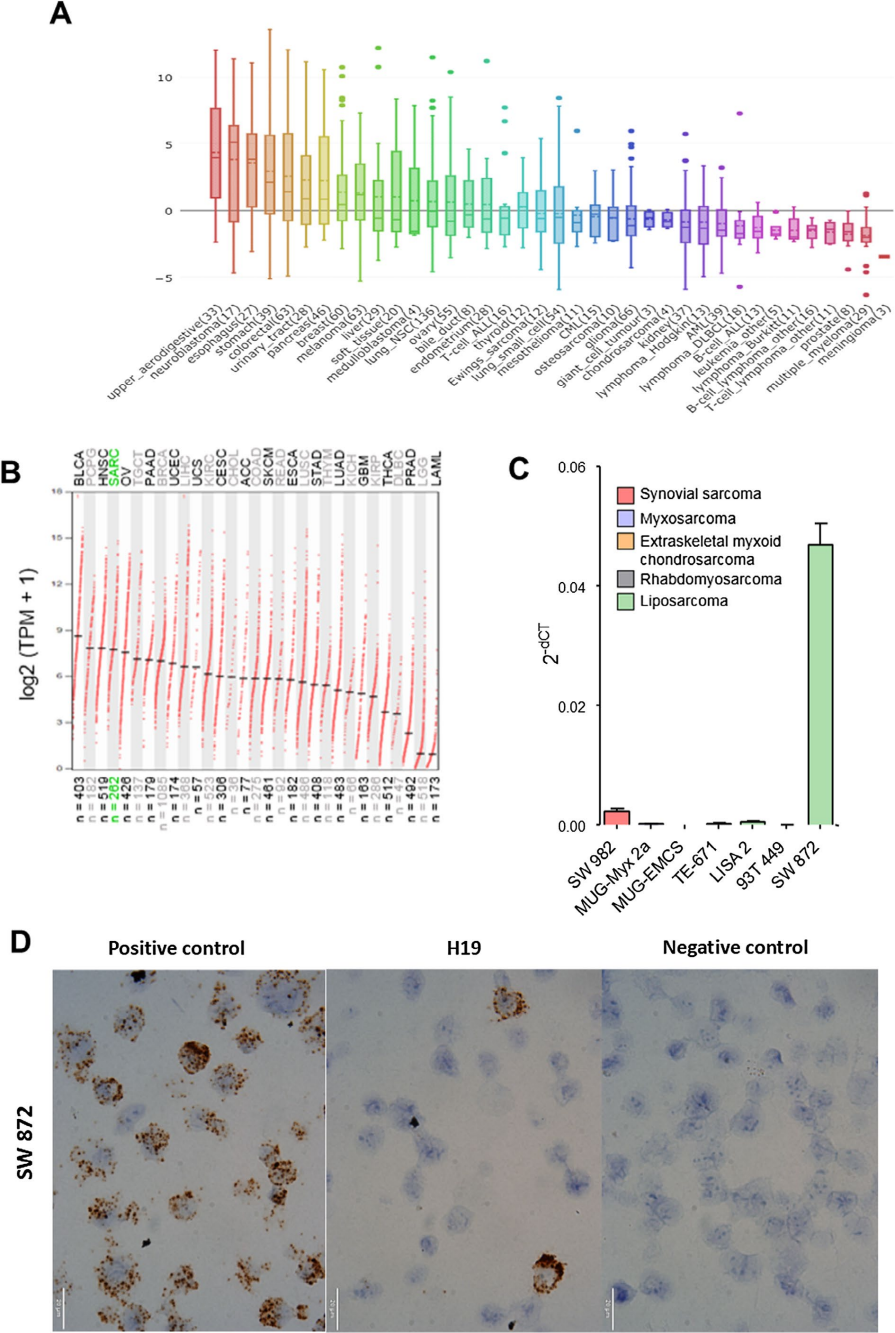
H19 expression in different sarcoma subtypes. (A) H19 RNA‐seq expression data across cell lines from 38 different cancer types derived from the publicly available CCLE database. (B) H19 RNA‐seq expression data from 31 cancer types in tumor tissue derived from TCGA and GTEx data (SARC = sarcoma; TMP = Transcript Per Million). (C) The expression of H19 in 7 different sarcoma cell lines was measured by qRT‐PCR and normalized to the housekeeper genes GAPDH and U6 (*n* = 3; mean ± SD). (D) Representative pictures of RNA in situ hybridization of H19 in the liposarcoma cell line SW872 showing a heterogenous expression pattern.

Whether and to what extent the lncRNA H19 is expressed in the heterogeneous group of sarcomas is largely unknown. Therefore, in the first screening step, we compared the occurrence and expression levels of H19 between different cancer types by using publicly available RNA‐seq data provided by the Broad Institute Cancer Cell Line Encyclopedia (CCLE) that comprises expression data of cell lines originating from 38 different cancer types. These included four sarcoma subtypes, namely Ewing sarcoma (12 cell lines), osteosarcoma (10 cell lines), chondrosarcoma (4 cell lines), and soft tissue sarcoma (20 cell lines). The highest H19 expression level was found for gastrointestinal cancers, whereas the lowest levels were depicted for hematological malignant disorders. In the group of sarcoma cell lines, the average expression level of H19 of all four subtypes was found to lie in the medium range compared to the included cancer types. Soft tissue sarcoma cell lines lie in the upper half of all cancer cell line groups (Figure 1A). In order to compare H19 expression from cell lines and tumor tissue samples, data from The Cancer Genome Atlas Program (TCGA) and the Genotype‐Tissue Expression (GTEx) project were analyzed using the online tool GEPIA [32]. Data from this platform showed that among 31 cancer types, soft tissue sarcomas (*n* = 262) had the fourth‐highest expression levels of H19 (Figure 1B). In the third step, we analyzed the expression of H19 in 7 different sarcoma cell lines using RT‐qPCR. These included a synovial sarcoma cell line (SW982), a myxosarcoma cell line (MUG‐Myx 2a), an extraskeletal myxoid chondrosarcoma cell line (MUG‐EMCS), a rhabdomyosarcoma cell line (TE‐671), and three different liposarcoma cell lines (LISA 2, 93T 449, SW872). The highest expression level was detected in the liposarcoma cell line SW872, whereas all other cell lines exhibited considerably lower or even undetectable levels of H19 (Figure 1C). To confirm H19 expression in the cell lines by an independent method, RNA in situ hybridization on cyto‐slides with the respective cell line SW872 confirmed and showed an expression pattern varying between cells (Figure 1D).

### 3.2 Evaluation of H19 as a Prognostic Biomarker

**FIGURE 2:**
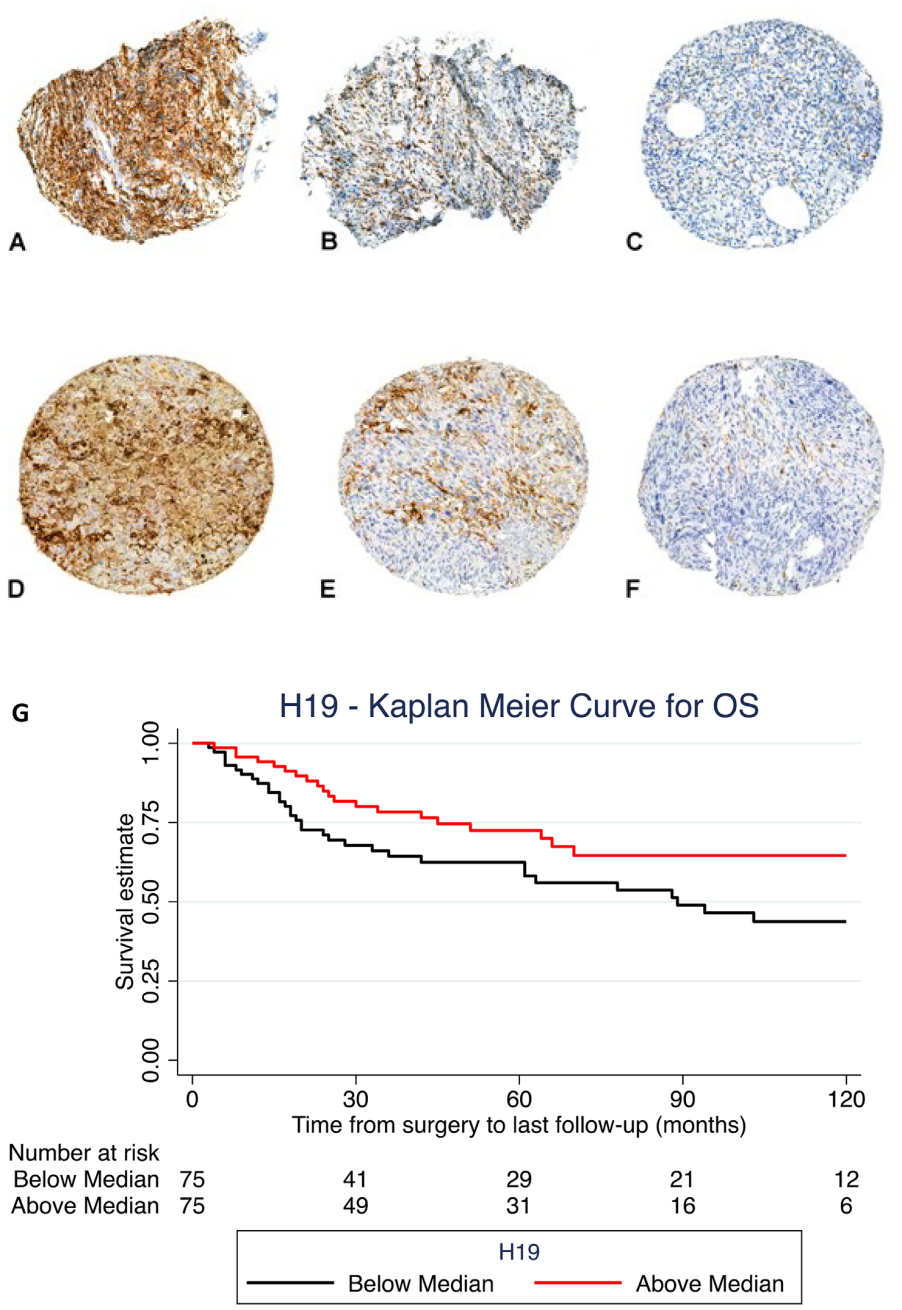
Representative pictures of RNA in situ hybridization staining of the H19 transcript expression in Tissue Microarray cores: (A) High expression in undifferentiated pleomorphic sarcoma, (B) medium expression in myxofibrosarcoma, (C) low expression in low‐grade fibromyxoid sarcoma. (D) High, (E) medium, and (F) low H19 expression in leiomyosarcoma. (G) Kaplan–Meier curve for survival cut at median H19 expression in a large cohort of soft tissue sarcoma patients (*n* = 150). Higher H19 expression is associated with improved survival.

To test H19 expression as a prognostic biomarker in soft tissue sarcoma, we used a large cohort of patients and applied RNA in situ hybridization for H19 on TMA slides (Figure 2A–F).

Tissue microarrays were assembled from FFPE tissue blocks processed for routine histopathological reporting (10% NBF fixed). Each tumor was represented by five tumor cores on the TMA (tissue core diameter 0.6 mm). Five TMAs were subjected to staining as described below: One TMA contained liposarcomas (225 cores/42 patients), two TMAs represented myxofibrosarcomas (MFS) (510 cores/95 patients), one TMA contained high‐grade sarcomas (defined by high cellular pleomorphism) (401 cores/63 patients), encompassing the following entities: Pleomorphic sarcoma (PS), dedifferentiated and pleomorphic liposarcoma, embryonal rhabdomyosarcoma (ERMS), myxoinflammatory fibroblastic sarcoma, (MFS) malignant peripheral nerve sheet tumor, low‐grade fibromyxoid sarcoma (LGFMS), leiomyosarcoma (LS), angiosarcoma, pleomorphic rhabdomyosarcoma, one TMA consisted of a broad array of sarcomatous entities (420 cores/79 patients), entities: synovial sarcoma (SS), MFS, extraskeletal myxoid chondrosarcoma, alveolar soft part sarcoma (ASPS), myxoid liposarcoma (MLS), dermatofibrosarcoma protuberans (DFSP), leiomyosarcoma (LMS), undifferentiated pleomorphic sarcoma (UPS), myxofibrosarcoma (MFS) and one TMA consisted of translocation sarcomas (275 cores/52 patients, entities: DFSP, LGFMS, MFS, ASPS, ERMS, MLS, ERMS, rhabdomyosarcoma, extraskeletal myxoid chondrosarcoma, malignant melanotic schwannoma, clear cell sarcoma, malignant mesenchymoma).

Overall, 150 patients (*n* = 79; 52.7% males) could be included in this cohort, with a mean age of 60.3 ± 17.8 years at the time of sarcoma diagnosis. Median follow‐up was 42.0 months (IQR: 19.0–89.0 months). Soft tissue sarcomas were most frequently located in the lower limbs (*n* = 102; 68.0%), and the mean tumor size was 8.9 ± 5.3 cm (Table 1). Median H19 expression was 23.0 [IQR: 6.3–84.5; range: 0.03–3777.2]. H19 expression was significantly higher in the upper limb location compared to the lower limb location (*p* = 0.012; Table 2). For other factors such as gender, grade, and histology, no significant difference in H19 expression was found. Furthermore, there was no significant association between H19 expression and patient age (*F*(1,148) = −0.37; *p* = 0.861), or tumor size (*F*(1,146) = 2.41; *p* = 0.740).

**TABLE 1:** Descriptive analysis of entire cohort (*n* = 150). Numbers are given with valid percentages.

Male gender	79/150 (52.7%)
Age at surgery (in years, mean ± SD)	60.3 ± 17.8
Tumor location	
Upper limb	32/150 (21.3%)
Lower limb	102/150 (68.0%)
Trunk	16/150 (10.7%)
Margins – R1/2	36/150 (24.0%)
Histology	
Leiomyosarcoma	17/150 (11.3%)
Myxofibrosarcoma	59/150 (39.4%)
MFH/UPS	17/150 (11.3%)
Liposarcoma	21/150 (14.0%)
Synovial sarcoma	10/150 (6.7%)
Others	26/150 (17.3%)
Grading	
G1	13/141 (9.2%)
G2	26/141 (18.4%)
G3	102/141 (72.3%)
Deep location	110/149 (73.8%)
Tumor size (in cm; mean ± SD)	8.9 ± 5.3
Adjuvant CTX – yes	18/150 (12.0%)
Adjuvant RTX – yes	85/143 (59.4%)
Local recurrence – yes	17/150 (11.3%)
Distant metastasis – yes	61/150 (40.7%)
Outcome	
Alive without disease	72/150 (48.0%)
Alive with disease	23/150 (15.3%)
Dead of disease	38/150 (25.4%)
Dead due to other causes	9/150 (6.0%)
Dead due to unknown causes	8/150 (5.3%)

**TABLE 2:** Association between H19 expression and clinical variables.

	H19 expression, median [IQR]	*p*
Gender		
Male	22.7 [6.7–79.0]	0.845
Female	23.0 [5.6–88.9]	
Tumor location		
Upper limb	36.9 [24.2–157.4]	**0.012** *
Lower limb	15.1 [3.9–63.1]	
Trunk	52.4 [6.7–362.0]	
Margins		
R0	23.3 [6.2–88.9]	0.424
R1/2	17.4 [6.7–41.9]	
Histology		
Leiomyosarcoma	9.0 [2.9–42.8]	0.321
Myxofibrosarcoma	28.7 [9.2–178.2]	
MFH/UPS	17.4 [8.7–51.9]	
Liposarcoma	35.0 [10.4–88.9]	
Synovial sarcoma	29.3 [11.3–76.0]	
Others	17.6 [2.3–94.0]	
Grading		
G1	11.4 [6.7–94.0]	0.773
G2	27.5 [10.4–79.0]	
G3	23.1 [6.2–78.3]	
Depth		
Superficial	24.9 [6.3–51.9]	0.644
Deep	22.8 [6.2–88.9]	
Adjuvant CTX		
No	23.0 [6.2–80.8]	0.843
Yes	24.9 [6.7–120.6]	
Adjuvant RTX		
No	12.3 [4.6–69.8]	0.370
Yes	28.6 [6.5–85.9]	

*Note:* Bold Values: The software programm used is not offering the output of exact *p*‐value when the value is below < 0.001.

* Kruskal–Wallis test: upper limb vs. lower limb: *p* = 0.007; upper limb vs. trunk: *p* = 0.264; lower limb vs. trunk: *p* = 0.120.

Cut at the median, a marginally positive impact of high H19 expression towards improved overall survival (OS) was found in the univariate Cox‐regression analysis (HR: 0.564; 95% CI: 0.324–0.985; *p* = 0.044; Table 3; Figure 2G). Large tumor size (*p* = 0.004) and advanced patient age at initial diagnosis (*p* < 0.001) were associated with worse OS. With leiomyosarcoma as a reference, the histological subtype liposarcoma showed a positive association with improved OS (*p* = 0.030; Table 3).

**TABLE 3:** Univariate Cox‐regression analysis for overall survival.

Univariate	HR	95% CI		*p*
Overall survival		Lower	Upper	
H19 expression				
Below median	1			**0.044**
Above median	0.564	0.324	0.985	
Gender				
Male	1			0.941
Female	1.021	0.595	1.751	
Mean age at surgery (in years)	1.041	1.022	1.061	**< 0.001**
Tumor location				
Upper limb	1			
Lower limb	1.098	0.545	2.215	0.793
Trunk	1.594	0.604	4.207	0.346
Margins				
R0	1			0.200
R1/2	0.625	0.305	1.281	
Histology				
Leiomyosarcoma	1			
Myxofibrosarcoma	0.621	0.272	1.414	0.256
MFH/UPS	2.215	0.887	5.532	0.089
Liposarcoma	0.264	0.079	0.879	**0.030**
Synovial sarcoma	1.126	0.368	3.445	0.836
Others	0.375	0.123	1.147	0.085
Grading				
G1	1			
G2	1.412	0.339	5.078	0.694
G3	2.478	0.767	8.005	0.129
Depth				
Superficial	1			0.274
Deep	1.449	0.746	2.816	
Mean tumor size (in cm)	1.064	1.020	1.111	**0.004**
Adjuvant CTX				
No	1			0.583
Yes	0.800	0.361	1.775	
Adjuvant RTX				
No	1			0.176
Yes	0.683	0.393	1.187	

*Note:* Bold Values: The software programm used is not offering the output of exact *p*‐value when the value is below < 0.001.

In multivariate analysis, only advanced patient age (*p* < 0.001) and large tumor size (*p* = 0.002) remained independently associated with worse OS, irrespective of H19 expression (HR: 0.655; 95% CI: 0.367–1.170; *p* = 0.153) or histology (*p* > 0.05; Table 4).

**TABLE 4:** Multivariate Cox regression analysis for overall survival.

Multivariate	HR	95% CI		*p*
Overall survival		Lower	Upper	
H19 expression				
Below median	1			0.153
Above median	0.655	0.367	1.170	
Mean age at surgery (in years)	1.045	1.022	1.069	**< 0.001**
Histology				
Leiomyosarcoma	1			
Myxofibrosarcoma	0.549	0.237	1.271	0.161
MFH/UPS	1.552	0.611	3.946	0.356
Liposarcoma	0.337	0.092	1.227	0.099
Synovial sarcoma	2.928	0.902	9.511	0.074
Others	0.900	0.283	2.861	0.858
Mean tumor size (in cm)	1.078	1.030	1.133	**0.002**

*Note:* Bold Values: The software programm used is not offering the output of exact *p*‐value when the value is below < 0.001.

### 3.3 RNA‐Directed KnockDown of H19 Leads to Decreased Cellular Growth and Increased Apoptosis in Certain Types of Soft Tissue Sarcoma

**FIGURE 3:**
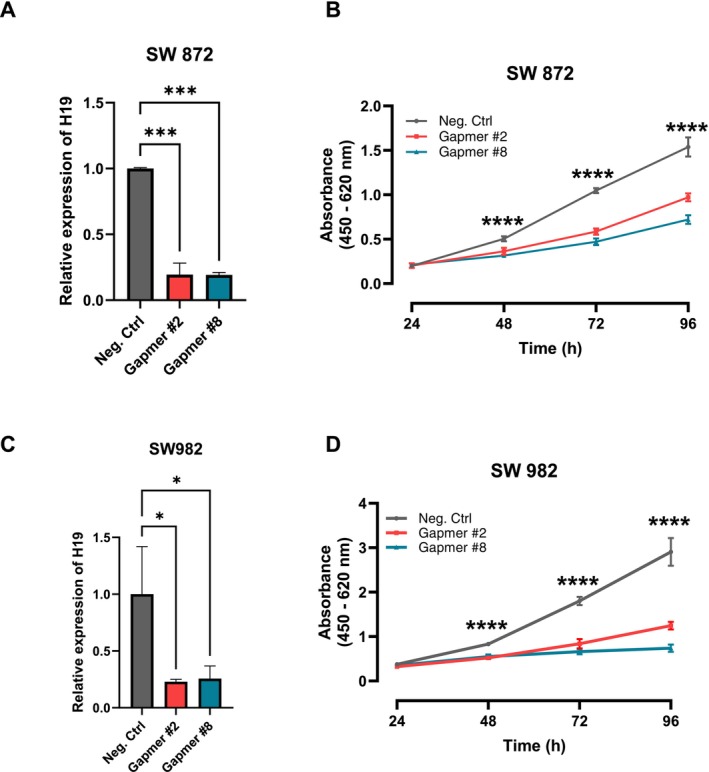
Targeting H19 by Gapmer‐mediated knock‐down leads to a reduction of cellular growth. (A, C) Quantification of knock‐down efficiency of H19 in two sarcoma cell lines after treatment with specific Gapmers against H19 or negative control Gapmer A 48 h after transfection on RNA level via real‐time PCR (*n* = 3; mean ± SD; *p < 0.05, ***p < 0.001). (B, D) Cellular growth after H19 knockdown in two sarcoma cell lines. WST‐1 cellular growth assay in sarcoma cell lines over 96 h under control conditions (Negative Control Gapmer A) or after Gapmer‐mediated knockdown of H19 (*n* = 6, mean ± SD; ****p < 0.001).

**FIGURE 4:**
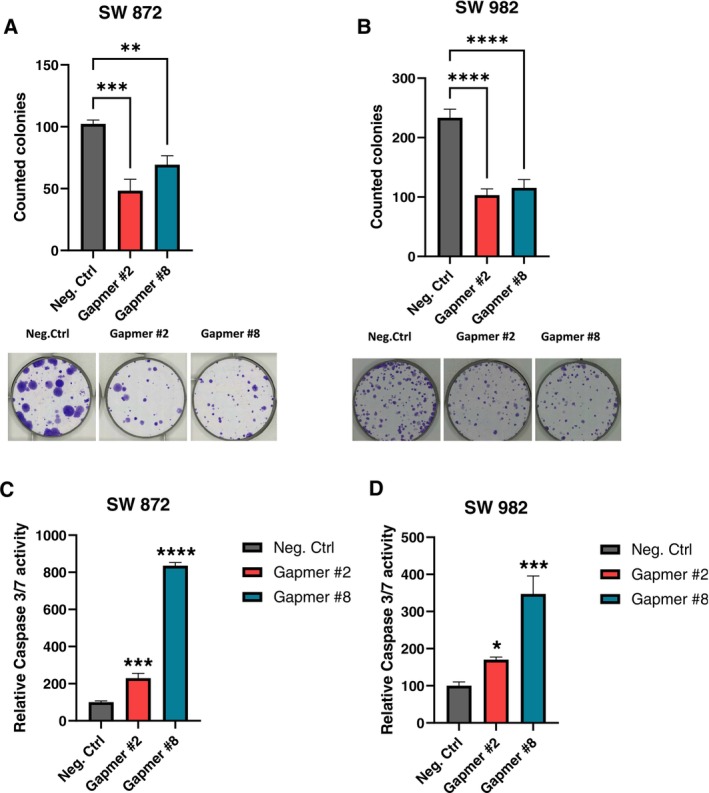
In vitro phenotypic characterization of H19 silencing in two sarcoma cell lines. (A, B) Clony formation assay in two sarcoma cell lines. The bar graphs on top represent absolute colony numbers under control conditions (Negative Control Gapmer A; gray bars) or after Gapmer‐mediated H19 knockdown (red and blue bars). *n* = 3, ±SD. **p < 0.01, ***p < 0.001, ***p < 0.0001. The bottom panels depict corresponding representative pictures. (C, D) Caspase 3/7 assay under control conditions (Negative Control Gapmer A, gray bars) or upon Gapmer‐mediated knockdown of H19 (red and blue bars) 48 h after transfection (*n* = 3, mean ± SD; ***p < 0.001, ****p < 0.0001).

To investigate the relevance of H19 in soft tissue sarcoma as a potential therapeutic target, we established a gene knock‐down approach by using Gapmers directed against H19 in two independent soft tissue sarcoma cell lines (synovial sarcoma SW982 and liposarcoma SW872). These cell lines displayed the highest H19 expression levels in the cell line panel screen shown in Figure 1C. The successful knockdown of H19 (Figure 3A,C) significantly reduced cellular growth in both cell lines (Figure 3B,D). This result was independently confirmed by a colony formation assay showing a significantly lower number of colonies formed in cells with decreased H19 expression levels (Figure 4A,B). To examine whether the decreased cellular growth might be caused by activation of apoptosis, we measured the caspase 3/7 activity 48 h after H19 silencing. In both the SW872 and SW982 cells, the knockdown of H19 led to increased caspase activity (Figure 4C,D).

## 4 Discussion

In the present study, we demonstrate that the lncRNA H19 is differentially expressed across soft tissue sarcoma subtypes and that, in univariate analysis, low levels of H19 are associated with poor survival in soft tissue sarcoma. However, when adjusted for traditional prognostic factors such as age and tumor size, H19 did not prevail as an independent prognostic factor in soft tissue sarcoma patients.

Next, we sought to determine whether the expression of H19 in soft tissue sarcoma cells might provide a therapeutic target. Using Gapmers, we successfully reduced the expression of H19, which led to decreased cellular growth corroborated by increased apoptotic activity.

Previous in vivo studies have evaluated the prognostic and biological influence of H19, mainly in osteosarcoma and rhabdomyosarcoma, and arrived at partially divergent conclusions regarding the functional role of H19. Findings by Lee et al. in osteosarcoma align with our prognostic in vivo data, demonstrating that low H19 expression promotes sarcoma pathogenesis [33]. The authors identified H19 as critical to osteogenic dedifferentiation in osteosarcoma. Zhao et al. reported that H19 overexpression is associated with poor overall survival in osteosarcoma patients and positively associated with the formation of distant metastases [30].

In vitro, targeting H19 by RNA interference (RNAi) has been demonstrated to reduce osteosarcoma cell migration and invasion by modulating the NF‐κB pathway [30]. On the other hand, H19 was also reported to increase proliferation in osteosarcoma cells by acting as a competing endogenous non‐coding RNA sponging microRNAs [34]. This supports our in vitro data in two analyzed cell lines but contrasts with in vivo data from the prevailing study and those of others as mentioned above and could, among other factors, pertain to cell culture data not fully replicating the corresponding sarcoma biology in vivo.

Furthermore, H19 has also been demonstrated to interact with proteins involved in DNA damage response and repair, indicating a regulatory role in genomic integrity [35]. Compared to osteosarcoma, the role of H19 in rhabdomyosarcoma has been less extensively studied. A study by Hao et al. suggests a tumor suppressive role for H19 in rhabdomyosarcoma, which was later confirmed by Casola et al., showing that H19 expression is suppressed in this tumor entity [24, 36]. Of note, rhabdomyosarcomas are associated with genetic alterations in a specific chromosomal region (11p15) that harbors a cluster of imprinted genes, including H19 [37, 38]. The aberrant methylation and subsequent loss of heterozygosity in this region can lead to the inactivation of H19, contributing to rhabdomyosarcoma development [24, 27]. These findings underscore the potential prognostic and functional role of H19 in the progression of osteosarcoma and rhabdomyosarcoma, although with heterogeneous functional assertions. To the best of our knowledge, only one dataset of the REGOSARC study has explored the prognostic and predictive role of H19 in soft tissue sarcoma patients with tissue microarrays (TMA) and RNA in situ hybridization techniques [31]. This study evaluated the association between H19 expression and overall survival in sarcoma patients, indicating a potential prognostic role for H19 as a biomarker. However, due to the limited sample size of the trial, the prognostic value did not reach statistical significance. The lack of significance may be due to the heterogeneity of the soft tissue sarcoma subtypes included in the analysis and the highly selected patient cohort of regorafenib‐treated metastatic soft tissue sarcoma patients. H19 was detected in 18% (*n* = 134) of tumor tissue samples with varying expression levels among the different soft tissue sarcoma subtypes, with the highest proportion observed in synovial sarcoma tissue (41%; *p* = 0.02). The higher frequency of H19 in synovial sarcoma suggests its potential as a specific biomarker and potential therapeutic opportunity for this subtype. As the survival analysis across all soft tissue sarcoma subtypes did not show a significant association between H19 expression and overall survival, focusing on larger cohorts of synovial sarcomas might uncover a significant prognostic impact of H19 expression on patient outcomes [31]. These observations underline that sarcoma is not a uniform entity and that there is considerable complexity and heterogeneity between different sarcoma subtypes regarding H19. Our study is limited by inter‐patient variability, histological diversity, and sample size, which may have masked subtle differences in H19 expression between tumor sites. Alternatively, given that H19 is regulated by broader oncogenic pathways and tumor microenvironmental factors rather than anatomical site alone [39, 40, 41], its expression may not significantly differ between tumor sites.

The above‐discussed studies show H19 as a potential biomarker and pharmacological target, although its role in tumorigenesis remains conflicting. While several in vivo studies, despite their limitations, suggest that H19 acts as a tumor suppressor [33, 36, 42], various in vitro experiments, including ours, point to a possible oncogenic function for H19 [18, 19, 23]. It is well known that long lncRNAs are highly versatile and act context‐dependently. It is conceivable that H19 indeed exerts opposing functional properties (tumor suppressive vs. oncogenic) in vivo compared to in vitro. Different functions may depend on the sarcoma subtype and the functional states of neoplastic cells within currently defined histological entities. Differences in the experimental systems and study designs are also plausible explanations for the current discrepant findings. Moreover, one needs to consider that the association of H19 with clinical outcomes has been assessed among patients receiving therapies, a context that has not been addressed in our study.

Our study is limited by its retrospective nature while including different sarcoma subtypes. In addition, the patients included might also have concurrent co‐morbidities and were subject to different treatment algorithms. In conclusion, our study provides evidence that H19 expression levels vary between different soft tissue sarcoma subtypes and provides unprecedented experimental evidence that H19 might be a valuable target for RNA‐targeting drugs to tackle H19 in soft tissue sarcomas. Another limitation of our study could be the inclusion of histologically diverse sarcoma subtypes, such as rhabdomyosarcoma, schwannoma, and mesenchymoma, which exhibit distinct biological features and may influence the generalizability of our findings across soft tissue sarcomas.

## Author Contributions

**Stephan Jahn:** writing – review and editing, conceptualization, methodology, investigation. **Katarina Krajina:** writing – review and editing, conceptualization, methodology, investigation. **Maria Anna Smolle:** data curation, formal analysis, writing – review and editing, conceptualization. **Dimyana Neufeldt:** writing – review and editing, conceptualization, methodology, investigation. **Katharina Jonas:** writing – review and editing, conceptualization, methodology, investigation. **Beate Rinner:** writing – review and editing, conceptualization, methodology, investigation. **Kevin Mellert:** writing – review and editing, conceptualization, investigation, methodology. **Maxim Noeparast:** writing – review and editing, conceptualization, methodology, investigation. **Martin Trepel:** writing – review and editing, conceptualization. **Joanna Szkandera:** data curation, formal analysis, writing – review and editing, conceptualization. **Martin Pichler:** data curation, formal analysis, writing – review and editing, conceptualization, methodology, investigation, supervision. **Bernadette Liegl‐Azwanger:** writing – review and editing, conceptualization, methodology, investigation, supervision.

## Disclosure

Permission to reproduce material from other sources: Data from a publicly available database (https://portals.broadinstitute.org/ccle) was queried for analyses.

## Ethics Statement

Medical University of Graz's local ethics commission approved this study (No. 29‐205 ex 16/17).

## Consent

The authors have nothing to report.

## Conflicts of Interest

The authors declare no conflicts of interest.

## Data Availability

The data that support the findings of this study are available in Broad Institute at https://portals.broadinstitute.org/ccle. These data were derived from the following resources available in the public domain: Broad Institute, https://portals.broadinstitute.org/ccle.
